# Peer‐Delivered Outreach With Rapid Treatment Pathways for Hepatitis C Testing and Treatment Among Unhoused People

**DOI:** 10.1111/jvh.70085

**Published:** 2025-09-15

**Authors:** Gabriele Vojt, Philippe Bonnet, Jennifer Scott, Emma Hathorn, Lisa Ellis, Sally Bufton, David Mutimer, Ryan Buchanan, Leila Reid, Danny Morris, Ahmed Elsharkawy

**Affiliations:** ^1^ National Institute for Health and Care Research (NIHR) Health Protection Research Unit in Evaluation and Behavioural Science University of Bristol Bristol UK; ^2^ Bristol Medical School University of Bristol Bristol UK; ^3^ Hepatitis C Trust London UK; ^4^ University Hospitals Birmingham NHS Foundation Trust Birmingham UK; ^5^ NHS England Hepatitis C Operational Delivery Network Birmingham West Midlands UK; ^6^ Faculty of Medicine University of Southampton Southampton UK

**Keywords:** community health workers, hepatitis C, ill‐housed persons, people who inject drugs, point‐of‐care testing

## Abstract

This service evaluation describes the co‐development of a peer‐led rapid hepatitis C virus (HCV) pathway to reach unhoused people. A trained and qualified peer worker visited homeless shelters in West Midlands, England, setting up test and treatment events and collaborating with local services and healthcare staff who also attended the sites. The peer worker offered point of care HCV antibody and ribonucleic acid (RNA) testing for individuals at risk of HCV, peer education and support before and during treatment. Viraemic individuals were offered immediate treatment prescribed by local HCV clinical specialist nurses who attended the homeless shelters with the peer worker. Among the 140 tested individuals, 72 people (51.4%) were HCV antibody positive and 42 (30.0%) were HCV RNA positive. All participants had a history of injecting drug use. The majority were male (75.0%), with a mean age of 39 years and of white ethnicity (89.4%). Treatment uptake was 100.0%, and known treatment completion was 92.3%. Treatment uptake within 2 weeks was 57.1%. Findings suggest that the co‐developed and peer‐led HCV test and treat pathway is promising in case finding, testing and treating marginalised, unhoused people.

## 1 Introduction

The aim of eliminating hepatitis C virus (HCV) by 2030 [1] has driven substantial innovation in HCV models of care. In England (UK), this has transformed the accessibility of HCV treatment for marginalised populations (e.g., people who inject drugs) in community and criminal justice. To reach HCV elimination goals, the UK strategy utilises a multi‐agency approach to develop innovative HCV care pathways that address regional needs and gaps in HCV service provision in HCV Operational Delivery Networks (ODN). This means HCV services constantly evolve, pilot and scale up innovative interventions and pathways. Central to HCV models of care are peer workers, who can exert powerful social influences across their (former) drug networks, increasing credibility and engagement when compared to healthcare and other professionals. In England, most ODNs work with the Hepatitis C Trust (HCT), a peer‐led organisation employing, training and providing supervision and support for peer workers [2]. Yet, in some regions of England, current models may not be optimal, and more people who inject drugs may need to access testing and treatment to meet elimination targets [3].

Point of Care (POC) testing can be used to detect exposure (antibody presence) to HCV and current infection through HCV Ribonucleic Acid (RNA) viral load testing. This enables staff to rapidly determine viraemia and initiate a treatment referral within 60 min [4]. The use of POC testing is associated with faster treatment initiation (i.e., within 14 days of diagnosis) and increased uptake among populations who use drugs. This is particularly important for unhoused people as they face high rates of HCV and multiple barriers to accessing HCV services.

In this paper, we report a pilot service evaluation of a co‐designed and peer‐delivered POC outreach HCV test and treatment pathway, specifically targeting populations who are houseless and are at risk of HCV.

## 2 Methods

### 2.1 Design

A quantitative retrospective pilot service evaluation of a co‐developed peer‐delivered outreach HCV testing and treatment pathway.

### 2.2 Participants

One hundred and forty unhoused people were tested. Table 1 provides further sample demographics.

### 2.3 Procedure

The Hepatitis C Trust (HCT), the regional West Midlands (Birmingham) NHS England HCV ODN, and local homelessness support services co‐developed a peer‐led rapid HCV test and treat pathway (see Appendix S1). This pathway was piloted in settings attended by unhoused people, for example, homeless shelters, soup kitchens, drug services, needle and syringe programmes, between November 2019 and February 2020. People were included in the pilot if they (1) resided in homeless shelters, reported to be without a fixed abode or sleeping on the street, (2) ever injected substances (currently and historically), or were currently on opioid substitution treatment and (3) were aged 18+ years. People were excluded from the pilot evaluation if they did not reside in a homeless shelter or slept on the streets, if they had never injected drugs and if they were aged under 18 years. Service staff introduced eligible people to the HCT peer worker who collected routine clinical data (e.g., history of drug and alcohol treatment, symptoms of liver disease, any allergies) and conducted HCV testing in two stages: (1) HCV antibody testing via a rapid HCV antibody POC test using oral fluids with results being available within 20 min. (2) If residents tested positive for HCV antibodies, or if they reported previous HCV infection, they were invited to test for HCV RNA using a GeneXpert HCV Viral Load Fingerstick blood test. The GeneXpert is highly specific (99%) and sensitive (99%) [5], test results are available within 60 min. People who tested negative for either antibodies or HCV RNA were given harm reduction advice by the HCT peer worker. Testing took place in private consultation rooms at participating services frequented by people who are houseless. All collected information was shared with the HCV clinical nurse specialist (CNS) team. Referrals for treatment initiation were either conducted immediately when HCV clinical nurse specialists were co‐located on site, or via telephone as part of the co‐developed HCV test and treat pathway. HCV DAA treatment was delivered by the HCT peer worker, either at the testing venue or a place of the individual's choice. Ongoing support with and beyond HCV diagnosis, testing and treatment were provided by the HCT peer worker.

### 2.4 Data Analysis

We conducted inferential analyses to describe the pilot population and test results using SPSS V30.

### 2.5 Ethics

This is a service evaluation and exempt from ethics approval (see Appendix S2) . It was reviewed and approved by an independent research advisory group, and a written data sharing agreement is in place with the participating ODN. The pilot described here is now routine service delivery in the ODN.

## 3 Results

### 3.1 HCV Testing Outcomes

Twenty‐one testing events took place in 15 individual settings. Individual settings were categorised as ‘hostel’, ‘street outreach’ (sleeping outside), ‘needle and syringe programme’ and ‘other’ (including services for unhoused sex workers and people frequenting drop‐in homeless services). Table 1 describes HCV test outcomes and patterns across individual testing settings.

**TABLE 1:** Participants' demographic background, HCV test outcomes and patterns across testing settings.

	All (*n* = 140)	Hostel (*n* = 48)	Street outreach (*n* = 38)	Needle and syringe programme (*n* = 46)	Other (*n* = 8)	*p*
Mean age (years) (SD)	38.8 (9.45)	38.5 (9.29)	37.7 (8.75)	41.0 (8.73)	38.7 (7.52)	0.340
Female	35 (25.0%)	6 (12.5%)	11 (28.9%)	16 (34.8%)	2 (25.0%)	0.083
Male	105 (75.0%)	42 (87.5%)	27 (71.1%)	30 (65.2%)	6 (75.0%)	
White ethnic background	129 (92.1%)	44 (91.7%)	35 (92.1%)	43 (93.5%)	7 (87.5%)	0.947
HCV antibody positive	72 (51.4%)	18 (37.5%)	26 (68.4%)	23 (50.0%)	5 (62.5%)	0.036
HCV RNA Positive	42 (30.0%)	15 (31.3%)	12 (31.6%)	12 (26.1%)	3 (37.5%)	0.047
HCV DAA treatment initiated within 30 days	31 (73.8%)	11 (73.3%)	7 (58.3%)	10 (83.3%)	3 (100.0%)	0.059
HCV DAA treatment initiated within 14 days	24 (57.1%)	11 (73.3%)	5 (41.7%)	5 (41.7%)	3 (100.0%)	0.220
HCV DAA treatment completed	39 (92.9%)	13 (86.7%)	11 (91.7%)	12 (100.0%)	3 (100.0%)	0.681

Abbreviations: DAA, Direct Acting Antiviral; HCV, Hepatitis C Virus; RNA, ribonucleic acid.

In hostels, while significantly fewer people tested HCV antibody positive (*n* = 18, 37.5%), the proportion who then tested positive for HCV RNA (*n* = 15/18) was significantly larger when compared to street outreach (*n* = 12/26) (*p* = 0.018) and needle and syringe programmes (*n* = 12/23) (*p* = 0.025). Treatment uptake was 100.0%. HCV treatment was initiated within a mean of 28.1 days (SD = 22.7) ranging from 8 to 102 days. The proportion of rapid (< 14 days) treatment recipients was highest in hostels. Four individuals (9.5%) engaged with the treatment pathway when followed up and supported individually by the peer worker (up to 14 weeks post the testing event).

## 4 Discussion

In this service evaluation, 140 unhoused people who inject(ed) drugs were tested for HCV in a range of outreach settings. Thirty percent tested positive for HCV RNA, and the majority (74%) commenced HCV treatment within 4 weeks of diagnosis. We demonstrate rapid treatment initiation through a co‐developed and peer‐led HCV test and treat pathway by overcoming barriers to HCV care with rapid diagnostic testing and treatment initiation, peer‐designed and led engagement, testing and treatment support. All HCV RNA positive individuals confirmed HCV treatment completion, except for three people whose completion is unknown as they were lost to follow up. This suggests that a co‐designed and peer‐delivered HCV testing and treatment package is well accepted. Our HCV RNA results (30%) are similar to the findings from peer‐led interventions targeting people who inject drugs (Norway [6] 34%, Queensland [7] 22%, Sydney [8] 27%) suggesting that peer work with different marginalised populations can reach large numbers of people with undiagnosed HCV. The potential of peer workers to support public health is further reflected in high treatment uptake; over 70% of people testing positive in our evaluation began treatment within 4 weeks of diagnosis, in line with 77% treatment uptake in Norway within 6 months, 84% in Queensland and 74% in Sydney within 7 days. Although the demographics of the studies differed, with less than half of participants in these studies coming from unhoused populations, this demonstrates the demand for HCV cure among healthcare‐excluded populations when offered in the right context and with peer support. Importantly, treating HCV in marginalised populations with high‐risk injecting behaviour (e.g., frequent sharing with a large network of individuals in restricted settings such as homeless shelters) via peers may have the greatest public health benefit by case finding highly networked individuals and preventing onward infection. These findings combined suggest that the synthesis of outreach care provision, peer‐facilitated engagement and follow‐up and the ability to share results rapidly via POC not only result in high identification of HCV RNA but also in completing HCV treatment.

## 5 Strengths and Limitations

We did not investigate the response rate among the wider target population, nor did we document SVR‐12 test results due to a lack of resources. We predominantly engaged white British individuals. Strengths include the co‐development of the HCV test and treat pathway and a multi‐disciplinary advisory team (healthcare professionals, third sector, academic researchers, hepatitis C consultants, peer workers) providing input to the interpretation of findings.

## 6 Future Research

Since this pilot, this co‐developed rapid HCV test and treat pathway has been implemented into routine HCV care across other NHS sites in England. Future research aims to report the number of people tested, treated and assessed at SVR12 through this pathway. Establishing treatment adherence and cost‐effectiveness of this pathway against a suitable control population is also important, as well as optimising referral effectiveness and examining the synergy and/or individual value POC testing and peer delivery models bring to HCV testing and treatment.

## 7 Conclusion

The described pathway enabled rapid HCV treatment initiation by overcoming barriers to care with rapid diagnostic testing, decentralisation of healthcare delivery, simplification of access and peer‐designed and led engagement, testing and treatment support. Combining peer expertise and knowledge with POC technology and supportive healthcare professionals is a promising HCV approach to engage, case find and treat unhoused people.

## Conflicts of Interest

The authors declare no conflicts of interest.

## Supporting information


**Appendix S1:** jvh70085‐sup‐0001‐AppendixS1.docx.

**Appendix S2:** jvh70085‐sup‐0002‐AppendixS2.pdf.

## Data Availability

Research data are not shared.
