# Supplementary material for: Peer‐Delivered Outreach With Rapid Treatment Pathways for Hepatitis C Testing and Treatment Among Unhoused People
Source: J Viral Hepat. 2025 Sep 15;32(10):e70085. doi: 10.1111/jvh.70085 (PMC12435099; doi:10.1111/jvh.70085)
Supplement: Supplementary file 1 — Appendix S1: jvh70085‐sup‐0001‐AppendixS1.docx. [file JVH-32-0-s002.docx]

Appendix 1

**Co-development of a rapid POC HCV test and treat pathway aimed at unhoused people**

*Setting.* Diverse settings where unhoused people may attend such as homeless shelters, soup kitchens, drug services and needle and syringe programmes in one health region, or Operational Delivery Network (ODN) in the UK. Across the National Health Service in the UK, all HCV testing and treatments are free of charge.

*Target population.* Unhoused people who inject(ed) drugs, who are out of treatment, or decline HCV testing at drug and needle syringe services.

*Key stakeholders.* The Hepatitis C Trust (HCT), the regional West Midlands (Birmingham) NHS England HCV Operational Delivery Network (ODN), and local homelessness support services. HCT is a peer-led non-governmental organisation employing peer workers to deliver HCV services to marginalised communities across the UK. HCT peer teams comprise paid and volunteer staff and work with every local health service and every prison in England, and in parts of Scotland and Wales. Peers work as part of local multi-disciplinary HCV teams, providing patient support, delivering peer education, staff training and testing for people at HCV risk. Peers attend induction HCV sessions, practical HCT skills training and shadow existing HCT peers as volunteers before they are employed at the HCT. All peers regardless of employment status receive clinical supervision, peer-to-peer mentorship and attend training courses around HCV, harm reduction, confidentiality and research skills. Those who are employed are line managed, supported to develop their competencies and are trained in conducting Dried Blood Spot Tests, and as part of the HCV pathway described here, in operating GeneXpert machines.

*Development of HCV pathway.* Key stakeholders co-developed a rapid test and treat HCV pathway for unhoused people, with bespoke documentation and referral options. The aim was to enable people diagnosed with HCV to initiate treatment within a few days and thereby reduce the time and risk of onward transmission. This was facilitated by removing the need for genotype testing through prescribing pangenotypic direct-acting antiviral (DAA) treatments to viremic individuals. The pathway required the HCT peer worker to be trained in operating a POC machine GeneXpert (Cepheid, California, USA) to determine HCV viral load. The peer worker also delivered HCV medications to patients or services where required. The pathway was initially tested in two services to identify facilitators and barriers to optimise procedures before piloting the pathway across the wider ODN region. Figure 1 outlines the pathway, including key set-up and collaboration steps.

**Figure 1** *Rapid POC HCV test and treat pathway aimed at unhoused people*


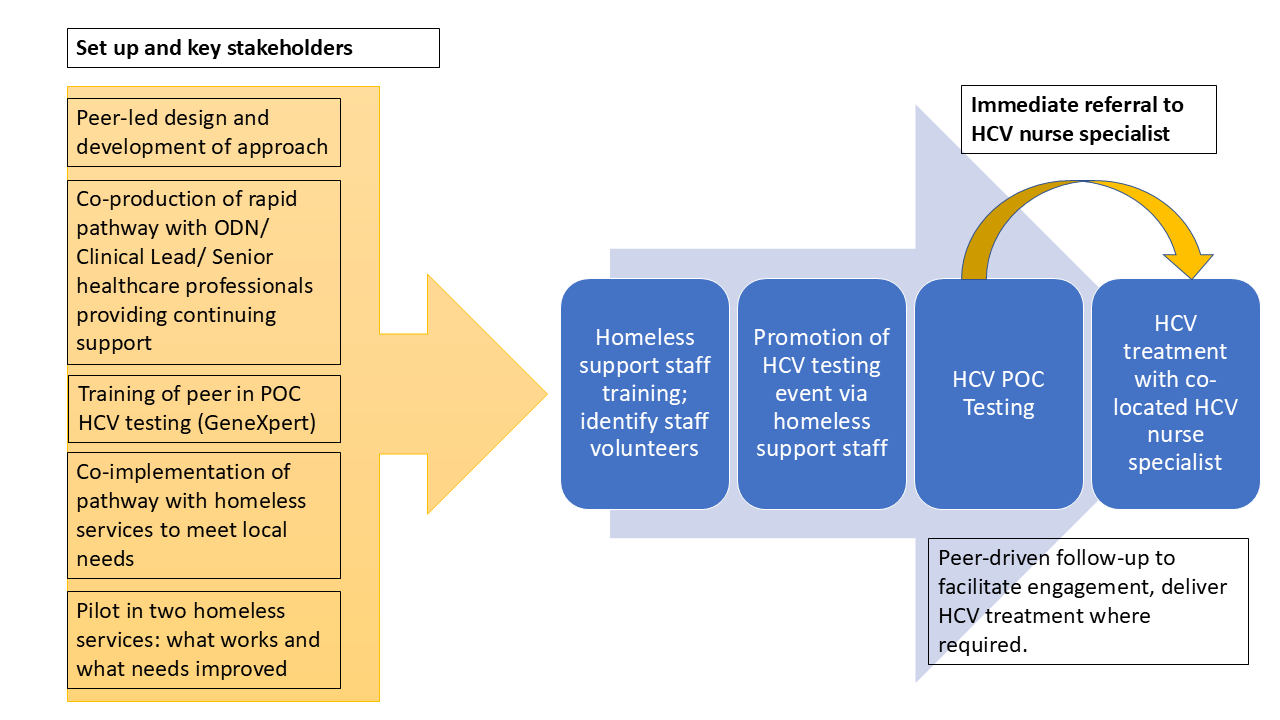


*Training.* The HCT peer worker delivered HCV education and awareness sessions to all staff in selected homelessness services. At these training events, interested staff members were asked, and volunteered, to promote and support the testing event.

*Promotion.* HCV testing event posters and flyers were designed by the peer worker to ensure information was accessible, credible and persuasive. These posters were then displayed in communal areas for a minimum of one week. In residential settings, the flyer was also posted under each resident’s door 24 hours before the event, by staff members. Each site promoted the event by providing free refreshments such as tea, coffee and biscuits to residents taking part; no other incentives were offered.

*POC HCV testing.* Staff introduced residents to the HCT peer worker, who then initiated a conversation about HCV, incorporating routine pre-testing and clinical assessments (e.g., history of injecting practices, alcohol and drug treatment, symptoms of liver disease, any allergies) and educational information (e.g., HCV transmission routes and risks). The HCT peer worker used their lived experiences of injecting drug use, HCV and wider life disadvantages to establish rapport, credibility and trust with the residents. The peer worker conducted all HCV testing. Testing was done in two stages: (1) all residents were offered testing via a rapid HCV antibody POC test using oral fluids with results being available within 20 minutes. (2) If residents tested positive for HCV antibodies, or if they reported previous HCV infection, they were invited to test for HCV RNA using a GeneXpert HCV Viral Load Fingerstick blood test. The GeneXpert requires a capillary whole-blood sample, collected from participants where their finger is pricked with a lancet. This removes the need for any venepuncture blood draw which can act as a significant barrier to HCV testing among people who inject(ed). The GeneXpert is highly specific (99%) and sensitive (99%), test results are available within 60 minutes. Across both testing stages, the peer worker maintained engagement with the resident and explained test results using accessible and empathic language, in particular normalising a positive test result and highlighting the ease, speed and curability of HCV with DAAs. Those residents who tested negative for either antibodies or HCV RNA were given harm reduction advice by the HCT peer worker. All collected information was shared with the HCV clinical nurse specialist (CNS) team.

*Referral.* Participants who tested positive for HCV RNA were immediately referred to the co-located HCV CNS on site. To facilitate this process, the ODN designed and approved a rapid referral form and approved prescribing of pangenotypic DAA treatment for all patients. When co-location with a HCV CNS was not possible, then referral via telephone was initiated using an adapted referral form.

*Treatment.* The peer worker offered door-to-door HCV treatment delivery. All service users were given the choice where they would like to receive their treatment, e.g., at the testing venue or elsewhere. HCV DAA treatments were provided in a single package for the full treatment course. While all HCV treatment prescriptions were aimed to be active within 14 days of diagnosis, the peer worker obtained additional contact information (e.g., community pharmacist) to minimise attrition before and during the treatment period. This is part of routine HCT service provision in England.
